# Ethnic and Geographic Differentiation of *Helicobacter pylori* within Iran

**DOI:** 10.1371/journal.pone.0009645

**Published:** 2010-03-22

**Authors:** Saeid Latifi-Navid, Seyed Ali Ghorashi, Farideh Siavoshi, Bodo Linz, Sadegh Massarrat, Tanya Khegay, Ali-Hatef Salmanian, Ali Akbar Shayesteh, Mohsen Masoodi, Koroush Ghanadi, Azita Ganji, Sebastian Suerbaum, Mark Achtman, Reza Malekzadeh, Daniel Falush

**Affiliations:** 1 Department of Bacteriology and Virology, National Institute of Genetic Engineering and Biotechnology, Tehran, Iran; 2 Digestive Disease Research Center, Shariati Hospital, Tehran University of Medical Sciences, Tehran, Iran; 3 Department of Microbiology, School of Biology, College of Sciences, University of Tehran, Tehran, Iran; 4 Department of Biochemistry and Molecular Biology, Pennsylvania State University, University Park, Pennsylvania, United States of America; 5 Uzbek Academy of Sciences, Institute of Immunology, Tashkent, Uzbekistan; 6 Department of Plant Molecular Biology, National Institute of Genetic Engineering and Biotechnology, Tehran, Iran; 7 Department of Internal Medicine, Imam Khomeini Hospital, Jundishapur University of Medical Sciences, Ahwaz, Iran; 8 Department of Internal Medicine, Gastrointestinal and Liver Disease Research Center (GILDRC), Iran University of Medical Sciences, Tehran, Iran; 9 Tropical and Infectious Disease Research Center, Hormozgan University of Medical Sciences, Bandar-Abbas, Iran; 10 Lorestan University of Medical Sciences, Khorramabad, Iran; 11 Department of Gastroenterology, Imam Reza Hospital, Mashhad University of Medical Sciences (MUMS), Mashhad, Iran; 12 Medizinische Hochschule Hannover, Institut für Medizinische Mikrobiologie und Krankenhaushygiene, Hannover, Germany; 13 Department of Microbiology, Environmental Research Institute, University College Cork, Cork, Ireland; University of Hyderabad, India

## Abstract

The bacterium *Helicobacter pylori* colonizes the human stomach, with individual infections persisting for decades. The spread of the bacterium has been shown to reflect both ancient and recent human migrations. We have sequenced housekeeping genes from *H. pylori* isolated from 147 Iranians with well-characterized geographical and ethnic origins sampled throughout Iran and compared them with sequences from strains from other locations. *H. pylori* from Iran are similar to others isolated from Western Eurasia and can be placed in the previously described HpEurope population. Despite the location of Iran at the crossroads of Eurasia, we found no evidence that the region been a major source of ancestry for strains across the continent. On a smaller scale, we found genetic affinities between the *H. pylori* isolated from particular Iranian populations and strains from Turks, Uzbeks, Palestinians and Israelis, reflecting documented historical contacts over the past two thousand years.

## Introduction

*Helicobacter pylori*, a major pathogen of the gastrointestinal tract, has been implicated in a wide spectrum of gastric disorders such as peptic and duodenal ulcerations as well as gastric cancer [1]. Genetic studies have established that the bacterium is highly diverse and that this diversity is geographically and ethnically structured [2], [3], [4], [5], [6]. For example, *H. pylori* from East Asia (e.g. Singapore and Korea) are distinct from those observed in Europe. Genetic diversity within *H. pylori* populations also tends to decrease with increasing distance from Africa, consistent with a similar but stronger cline observed in humans [4], [7], [8]. The biogeographic relationships within *H. pylori* are likely a result of intra-familial transmission combined with recycling within local communities [4], [9]. Unlike many other human pathogens, there has not been any long-range horizontal transmission from other species although humans donated Helicobacter to large cats some hundred thousand years ago [10]. Several putative and proven *H. pylori* virulence factors have also been described, including *cagA* and *vacA*, whose allele frequency have also been shown to vary by ethnic group [11].

To date, nine differentiated bacterial populations have been observed and there additionally is a strong gene frequency cline from Northern to Southern Europe [4], [6], [12]. This cline reflects the extensive mixing of two populations together, Ancestral Europe1 and Ancestral Europe2 (AE1 and AE2), which contribute approximately 30–70% of ancestry on average to each strain, with the average varying according to geographic location. It is not clear which population arrived first, but AE1 has higher frequency in Northern Europe, while AE2 is more common in Southern Europe. The names of these two ancestral populations reflect where genetic material from the two populations was first identified but in fact the two populations are found in purer form in Central Asia (AE1) and North East Africa (AE2). Populations with mixed AE1 and AE2 ancestry have also been found in the near East and in the Indian subcontinent [13].

While it has been shown two populations of *H. pylori* arrived in Europe and other parts of Eurasia at different times, it is currently not clear when or from where they arrived or how exactly these migrations relate to the peopling of the continent. In order to investigate these questions, we have sequenced *H. pylori* taken from Iranians with well-defined geographical and ethnic origin. Iran is a large and ethnically diverse country that sits at the crossroads between Europe, Asia and Africa. The country contains much of the Fertile Crescent where agriculture and civilization first developed. It has been estimated that 69% of the Iranian population currently harbour *H. pylori* infection [14] and show the frequent rate of development of duodenal ulcer [15] and gastric cancer [16], largely influenced by geographic and/or ethnic origin.

We have also compared unpublished sequences from *H. pylori* isolated from Uzbek and Tajik residents of Uzbekistan. Uzbekistan is one of the larger Central Asia states and borders Kazakhstan, Tajikistan, Kyrgyzstan and Turkmenistan. The latter is located between Uzbekistan and North Eastern Iran. Thus, the characterization of *H. pylori* population from both Iran and Uzbekistan may elucidate whether genetic exchange has occurred in this region of the world. The collection and genotyping of these strains from Uzbekistan will be described elsewhere.

## Materials and Methods

### Bacterial Isolates

**Figure 1:**
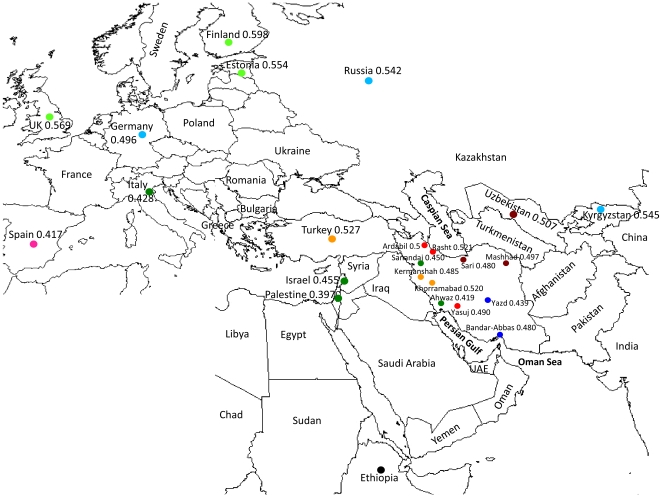
A detailed map with the labelled geographical locations that *H. pylori* populations were isolated. The proportion of AE1 nucleotides is indicated for each population. The proportion of AE2 ancestry is 1.0-the proportion of AE1 ancestry. The sample sizes for the non-Iranian samples were UK 7, Finland 10, Estonia 8, Russia 159, Kyrgystan 9, Turkey 19, Spain 71, Italy 7, Palestine 11, Israel 58, Germany 22, Uzbekistan (Uzbek) 55, Uzbekistan (Tajik) 17.

A total of 147 *H. pylori* isolates were obtained in 2007–08 from biopsy cultures from patients who were referred to the reference endoscopy units in different provinces of Iran. The biopsies were taken from patients who were of Iranian nationality, had the same place of birth and residency and gave the same ethnic/linguistic origin for both of their parents and for all four of their grandparents. In total, the strains were obtained from 7 defined ethnic groups within 11 districts of Iran (Figure 1, Table 1, Table S1).

**Table 1:** Description of Iranian strains.

Province	Area (No. strains)	Ethnic origin	Language family	Language high order subgroup	Language low order subgroup	Language	Geographical origin
**Ardabil**	Ardabil (21)	Azeri	Altaic	Turkic	Southern	Azerbaijani	Northwest of Iran
**Guilan**	Rasht (10)	Guilaki	Indo-European	Indo-Iranian	Iranian	Western	North of Iran
**Mazanderan**	Sari (13)	Mazanderani	Indo-European	Indo-Iranian	Iranian	Western	North of Iran
**Kurdistan**	Sanandaj (10)	Kurd	Indo-European	Indo-Iranian	Iranian	Western	West of Iran
**Kermanshah**	Kermanshah (12)	Kurd	Indo-European	Indo-Iranian	Iranian	Western	West of Iran
**Lorestan**	Khorramabad (10)	Lor	Indo-European	Indo-Iranian	Iranian	Western	West of Iran
**Kohgiluyeh & Boyerahmad**	Yasuj (7)	Lor	Indo-European	Indo-Iranian	Iranian	Western	Southwest of Iran
**Khorasan-Razavi**	Mashhad (18)	Fars	Indo-European	Indo-Iranian	Iranian	Western	Northeast of Iran
**Yazd**	Yazd (14)	Fars	Indo-European	Indo-Iranian	Iranian	Western	South of Iran
**Hormozghan**	Bandar-Abbas (10)	Fars	Indo-European	Indo-Iranian	Iranian	Western	South of Iran
**Khuzestan**	Ahwaz (22)	Arab	Afro-Asiatic	Semitic	Central	South	Southwest of Iran

Biopsies were transferred within 24 hr under cold chain to the *H. pylori* lab at the University of Tehran and were cultured on selective Brucella agar (Merck) containing blood under microaerobic conditions. Bacterial isolates were identified as *H. pylori* on the basis of Gram's stain, showing Gram-negative spiral forms, positive urease, oxidase and catalase tests as well as PCR amplification of *H. pylori* 16S rDNA [17]. Single colony isolation was performed in order to ensure that each strain consists of only a single genotype.

The Iranian strains were supplemented by sequences of 72 isolates from Uzbeks (55 isolates) and Tajiks (17) as well as published sequences from http://pubmlst.org/helicobacter. The published sequences were from 381 isolates from Europe and 51 from North-Eastern Africa and Ethiopia (Figure 1) and have been previously published by Falush et al. 2003 and Linz et al. 2007.

### Multilocus Sequence Typing (MLST)

We used the MLST scheme [2] in order to characterize the *H. pylori* strains using sequence analysis of the seven housekeeping genes composed of *atpA*, *efp*, *mutY*, *ppa*, *trpC*, *ureI* and *yphC*. DNA was extracted using a DNP™ kit (Cinagen Corporation, Iran). The primers listed at http://pubmlst.org/helicobacter were used for PCR amplification under the following conditions: 5 min of pre-denaturation at 96°C, 30 cycles of 40s at 96°C, 40s at 56°C (except *mutY* and *trpC* with an annealing temperature of 58°C and *ureI* and *ppa* with annealing temperatures of 52°C and 53°C, respectively), 40s at 72°C and a final incubation at 72°C for 7 min. PCR products were purified by Shrimp Alkaline Phosphatase/Exonuclease I (USB Corporation, USA) and were sequenced with both forward and reverse primers by using BigDye technology on an ABI3700XL DNA sequencer (Applied Biosystems).

All novel sequences generated in this study were deposited in GenBank database under the following accession numbers: GU444287-GU444433 (*atpA*), GU444434-GU444580 (*efp*), GU444581-GU444727 (*mutY*), GU444728-GU444874 (*ppa*), GU444875-GU445020 (*trpC*), GU445021-GU445166 (*ureI*) and GU445167-GU445313 ( *yphC*). The sequences are also deposited on the MLST website http://pubmlst.org/helicobacter.

### Data Analysis

All sequence traces of the *H. pylori* MLST loci were loaded into a BioNumerics v5.10 database (Applied-Maths, Sint Maartens-Latem, Belgium). The sequences were assembled, trimmed and edited in order to obtain the same sequence length according to the defined trimming patterns for all MLST loci. Neighbor-joining trees were obtained with MEGA v4 [18], using a distance matrix calculated using the Kimura 2 parameter model of sequence evolution. ClonalFrame analysis [19] was performed for the inference of bacterial microevolution with 100,000 iterations followed by a burn-in period of 50,000 iterations. The program estimates the clonal genealogy for a given set of DNA sequences by using a Bayesian-based neutral coalescent model.

The linkage model in STRUCTURE 2.2 [20] was used in order to identify the ancestral populations and assign ancestry proportions for each isolate. The model is based on the fact that closely linked alleles often inherited as a single unit from the same ancestral population. Each chunk is independently derived from a given number of bacterial populations, *k* with probability *q_k_*, where *q_k_* is the proportion of ancestry from each *k* population for each individual isolate. The program was run with *K = 2* by a Markov chain Monte Carlo (MCMC) simulation of 200,000 iterations followed by a burn-in period of 100,000 iterations.

We also studied the genetic structure of the populations by analyses of molecular variance (AMOVA) and pair-wise *F_ST_* using the software package Arlequin 3.1 [21]. The significance of the pair-wise *F_ST_* values were estimated by permutation analyses using 10,000 permutations with an assumption of no difference between the populations. The P-value was considered as the proportion of permutations resulting in the higher *F_ST_* value or equal to the observed one.

The study was approved by the ethics committee of the Digestive Diseases Research Center, Shariati Hospital, Tehran University of Medical Sciences, based on the ethical principles of human research and experimentation expressed in the Declaration of Helsinki. All hospitals involved in this study followed the approved ethical principles of DDRC committee. The subjects were all undergoing endoscopy as part of their treatment process. Informed consent for participation in the study was given by each subject in writing. The structured ethnic/linguistic questionnaire was completed for each subject by direct interview.

## Results and Discussion

**Figure 2:**
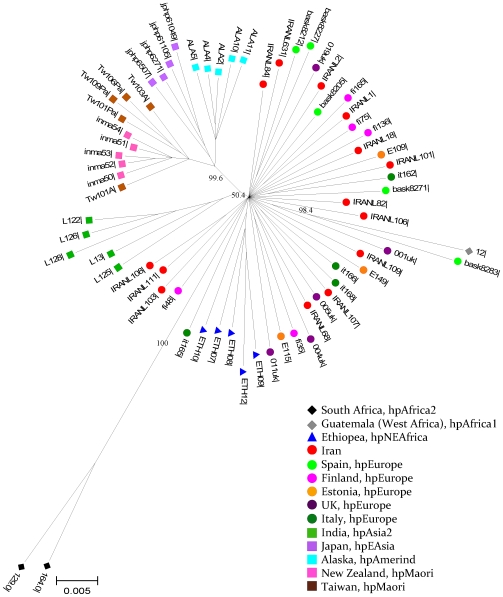
Neighbor-joining phylogenetic analysis of 68 worldwide *H. pylori* isolates using seven concatenated housekeeping gene sequences. The differentiated bacterial populations were recognized and called hpAfrica2, hpAfrica1, hspNEAfrica, hpEurope (hpEurope1 and hpEurope2), hpAsia2, hpEAsia, hpAmerind and hpMaori. The Iranian strains shared ancestral origins with the European counterparts. Each lineage is supported by a higher bootstrap value given at the corresponding branch. The strains were colour-coded according to the origins they were isolated.

For initial exploratory analysis, we assembled a dataset including 68 strains from each of the 9 previously described *H. pylori* populations and 14 Iranian strains (Figure 2). The Iranian isolates were intermingled with strains assigned to the hpEurope population. We also performed a phylogenetic analysis using the sequences of 330 *H. pylori* strains from Europe and North Africa as well as all 147 Iranian strains with ClonalFrame, a Bayesian method that takes into account any potential recombination events. This analysis again showed the Iranian strains to be intermingled amongst the hpEurope isolates, including isolates from Spain, the UK, Finland, Turkey and Italy (Figure S1). We were thus unable to identify clear population structure within the hpEurope population at the level of the individual strain.

The inability to differentiate strains at the individual level based on 7 MLST loci indicates a widespread sharing of DNA sequence polymorphisms amongst hpEurope strains from different locations. This sharing is consistent with bacteria rapidly circulating between different locations but is also consistent with a slower rate of spread if high levels of genetic variation amongst strains that is preserved within each geographic location over long time periods. We performed a hierarchical analysis of molecular variance to separate the total genetic variance into three covariance components as follows: within population (WP), among population/within group (AP/WG) and among groups (AG). The WP, AP/WG and AG components for molecular haplotypes explained 94.30%, 1.67% and 4.04% of variance, respectively. Thus, considerable variation is preserved at the population level.

**Figure 3:**
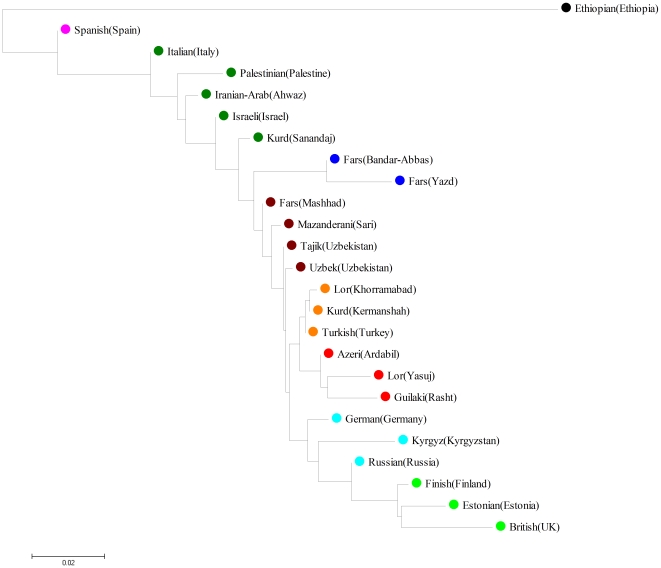
Neighbor-joining tree based on pairwise *F_ST_* values generated from hpEurope population.

In order to investigate signatures of genetic differentiation that are not visible at the individual level, we have calculated *F_ST_* between pairs of labelled populations (Table 2). This analysis showed that many pairs of populations are significantly differentiated and moreover provides an indication that there is geographical structure to this variation; for example most Iranian populations are differentiated from most European ones. In order to provide an overview of this diversity, we constructed a Neighbor-joining tree based on pairwise *F_ST_* values (Figure 3). This tree provided evidence for considerable geographic/ethnic substructure within the hpEurope population as a whole.

**Table 2:** Mean pair-wise *F_ST_* values between Iranian *H. pylori* populations and others with *HpEurope* signatures.

Population (No.)		1	2	3	4	5	6	7	8	9	10	11	12	13	14	15	16	17	18	19	20	21	22	23	24
**Azeri (21)**	**1**	**-**																							
**Guilaki (10)**	**2**	**0.011**	**-**																						
**Mazanderani (13)**	**3**	**0.007**	*0.048*	**-**																					
**Kurd (Sanandaj) (10)**	**4**	*0.021*	*0.038*	**0.004**	**-**																				
**Kurd (Kermanshah) (12)**	**5**	**0.000**	*0.028*	**0.010**	**0.004**	**-**																			
**Fars (Yazd) (14)**	**6**	*0.053*	*0.066*	*0.040*	*0.051*	*0.058*	**-**																		
**Fars (Mashhad) (18)**	**7**	**0.000**	*0.033*	**-0.004**	**0.005**	**0.005**	*0.023*	**-**																	
**Lor (Khorramabad) (10)**	**8**	**0.007**	**0.023**	**0.017**	**0.016**	**-0.001**	*0.070*	**-0.004**	**-**																
**Lor (Yasuj) (7)**	**9**	**0.007**	**0.025**	**0.019**	*0.066*	**0.022**	*0.067*	**0.020**	**0.029**	**-**															
**Arab (Ahwaz) (22)**	**10**	*0.039*	*0.076*	*0.031*	**0.008**	*0.023*	*0.055*	*0.019*	*0.029*	*0.065*	**-**														
**Fars (Bandar-Abbas) (10)**	**11**	**0.011**	**0.012**	**0.015**	**0.014**	*0.023*	**-0.005**	**-0.006**	**0.023**	**0.017**	*0.031*	**-**													
**British (7)**	**12**	*0.060*	*0.066*	*0.068*	*0.094*	*0.066*	*0.111*	*0.067*	*0.064*	*0.067*	*0.107*	*0.051*	**-**												
**Finish (10)**	**13**	*0.041*	*0.056*	*0.047*	*0.056*	*0.034*	*0.100*	*0.028*	*0.027*	*0.052*	*0.074*	*0.036*	**0.030**	**-**											
**Estonian (8)**	**14**	*0.043*	*0.072*	*0.059*	*0.086*	*0.058*	*0.097*	*0.046*	*0.053*	*0.060*	*0.094*	*0.045*	**0.037**	**0.015**	**-**										
**Spanish (71)**	**15**	*0.044*	*0.060*	*0.032*	*0.030*	*0.033*	*0.049*	*0.032*	*0.055*	*0.036*	*0.030*	*0.029*	*0.083*	*0.072*	*0.066*	**-**									
**German (22)**	**16**	*0.016*	*0.031*	*0.017*	*0.038*	*0.016*	*0.047*	*0.013*	*0.028*	**0.017**	*0.038*	**0.014**	*0.034*	*0.034*	*0.022*	*0.031*	**-**								
**Palestinian (11)**	**17**	*0.042*	*0.071*	*0.044*	**0.020**	*0.024*	*0.064*	*0.043*	*0.048*	*0.071*	*0.020*	*0.047*	*0.119*	*0.095*	*0.108*	*0.033*	*0.060*	**-**							
**Israeli (58)**	**18**	*0.026*	*0.056*	*0.016*	*0.016*	*0.016*	*0.025*	**0.007**	*0.021*	*0.044*	*0.010*	**0.008**	*0.067*	*0.045*	*0.044*	*0.023*	*0.019*	*0.027*	**-**						
**Italian (7)**	**19**	*0.051*	*0.078*	*0.040*	**0.021**	*0.029*	*0.050*	*0.024*	*0.038*	*0.090*	**0.016**	**0.021**	*0.105*	*0.087*	*0.085*	*0.027*	*0.039*	**0.022**	**-0.004**	**-**					
**Tajik (17)**	**20**	**0.002**	*0.021*	**0.003**	**0.015**	**-0.001**	*0.028*	**-0.006**	**0.002**	*0.023*	*0.017*	**0.001**	*0.059*	*0.038*	*0.048*	*0.027*	**0.007**	*0.032*	*0.008*	*0.024*	**-**				
**Uzbek (55)**	**21**	**0.005**	*0.031*	**0.007**	*0.017*	**0.003**	*0.042*	**0.001**	**0.011**	*0.029*	*0.032*	*0.014*	*0.064*	*0.031*	*0.041*	*0.044*	*0.017*	*0.042*	*0.024*	*0.043*	**-0.009**	**-**			
**Turkish (19)**	**22**	**0.000**	**0.009**	**0.000**	*0.017*	**-0.002**	*0.061*	**-0.001**	**-0.013**	*0.026*	*0.038*	**0.013**	*0.047*	*0.021*	*0.048*	*0.050*	*0.018*	*0.049*	*0.024*	*0.053*	**0.003**	*0.009*	**-**		
**Kyrgyz (9)**	**23**	*0.033*	*0.034*	*0.046*	*0.058*	*0.036*	*0.077*	*0.027*	*0.035*	*0.057*	*0.074*	*0.047*	*0.074*	*0.055*	*0.047*	*0.053*	*0.035*	*0.078*	*0.046*	*0.084*	*0.022*	*0.020*	*0.036*	**-**	
**Russian (159)**	**24**	*0.019*	*0.034*	*0.026*	*0.033*	*0.018*	*0.055*	*0.014*	*0.016*	*0.035*	*0.046*	*0.022*	*0.041*	**0.009**	*0.015*	*0.049*	*0.012*	*0.060*	*0.027*	*0.055*	*0.011*	*0.014*	*0.014*	*0.021*	**-**

Significant FSTs have been shown in italics (Significance Level = 0.0500).

The Iranian groups fall into five clusters, three of which also contain non-Iranian populations in the current dataset. The Iranian Arab population clustered between Palestinian and Israeli strains. Kurds, from Sanandaj in Northwest Iran also clustered close to this group. A second Kurd population from Kermanshah and Lor from Khorramabad, both in West Central Iran formed a quite distinct cluster together with strains from Turks. The two populations on the North Eastern border, in Sari and Mashhad, form a third cluster together with the Tajik and Uzbek populations from Uzbekistan.

**Figure 4:**
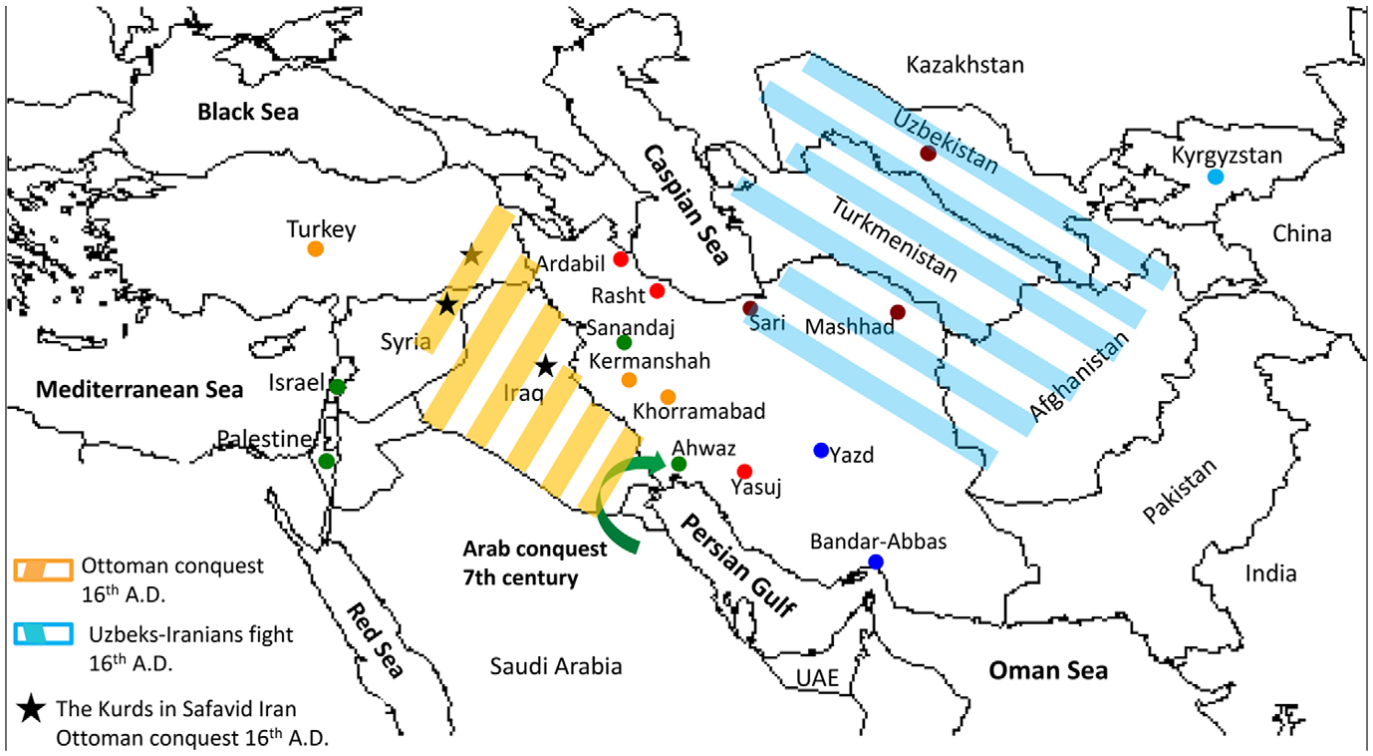
Detailed map highlighting historical events postulated to be responsible for similarities of Iranian isolates with those of neighboring countries.

These three trans-national clusters provide evidence for geographic and ethnic differentiation within Iran that reflects historical interactions with external populations (Figure 4). The bulk of the Iranian Arab population arrived in Iran in the 7th and 8th Centuries during the Islamic conquest of Persia and has kept its distinct identity and languages till the present day [22]. The Kurds in North Western Iran have had extensive historical contacts with Turkish Kurds and other Turks during several periods of history, including during the Ottoman Empire up to 1514, during the First World War and in the later part of the 20^th^ Century. The Uzbeks fought with the Iranian Safavid dynasty for control of areas of North Eastern Iran during the 16th Century [23], [24].

Two other clusters have currently only been found within Iran but might also share affinities with currently unsampled neighbouring populations. The most distinct is found in the two South Easterly populations, in Yazd and Bandar-Abbas, It would be interesting to establish whether this cluster is genetically similar to strains carried by people living within the Persian Gulf or with other populations further to the East. The final cluster is found in three Westerly populations, including two on the most Northerly part of Iran and could potentially be closely related to Armenian or Azerbaijani populations.

We used the linkage model of STRUCTURE to estimate the proportion of AE1 and AE2 ancestry for strains in each group (Figure 1). The variation in ancestry proportions amongst groups fell within the range that has already been seen within populations in Europe and the Middle East [6]. The overall proportion of AE1 in Iran is higher than for the sampled European populations at similar or slightly higher latitudes (i.e. Spain, Italy). The Southerly populations do have a slightly lower proportion of AE1 ancestry than the Northern ones, suggesting that there might also be a North-South cline, however the trend is weak and might be overwhelmed by the specific regional influences discussed above. The population with the lowest proportion of AE1 ancestry was the Iranian Arabs from Ahwaz who had 0.419 AE1 ancestry. This is comparable to the ancestry of the Palestinian and Israeli strains with whom they cluster in the FST tree (0.397 and 0.455 AE1 ancestry, respectively). The relatively low proportion of AE2 ancestry of the bulk of Iranian isolates, compared with Spain and Italy suggests that AE2 initially entered Eurasia not via the Middle East as has previously been suggested [6] but more probably via Southern Europe.

*Helicobacter pylori* from Iran are similar to others isolated from Western Eurasia and can be placed in the previously described HpEurope population [6]. HpEurope has been formed by the mixture of two distinct ancestral populations, AE1 and AE2, but in proportions that vary according to location. Iranian isolates are unexceptional, with approximately equal contribution from the two ancestral sources. For this reason it does not appear likely that either source came via Iran before spreading throughout Western Eurasia.

We found genetic affinities between the *H. pylori* isolated from particular Iranian populations and the strains from ethnically or geographically similar strains in nearby countries. This contrasts with a previous analyses based on human mtDNA and Y-chromosome data which, despite larger sample sizes and a larger number of nearby sampling locations, did not find similar clear associations [25]. These findings are concordant with similar results for strains sampled in Ladakh, where *H. pylori* proved more discriminatory than uniparental human genetic markers or a small microsatellite panel in distinguishing ethnic groups in the same geographic location on an individual by individual basis [26]. The high resolution of *H. pylori* to detect these local patterns, reflecting recent patterns of population movement, is a consequence of the slow transmission of the bacteria between ethnic groups and the fast rate of evolution of its sequence compared to human DNA.

## Supporting Information

Figure S1 Phylogeny of 330 worldwide H. pylori strains using ClonalFrame. The majority-rule consensus tree showed a very close relationship between Iranian strains and European counterparts. Most European isolates, especially from Spain, UK, Finland and Italy shared most recent common ancestor with Iranian ones in different sub-clades. The strains from NEAfrica were also grouped into two distinct clades. The strains were colour-coded according to the origins they were isolated.
(1.07 MB TIF)

Table S1 Sources of Iranian strains.
(0.06 MB XLS)
