# Supplementary figures and images for: Ethnic and Geographic Differentiation of Helicobacter pylori within Iran
Source: PLoS One. 2010 Mar 22;5(3):e9645. doi: 10.1371/journal.pone.0009645 (PMC2842290; doi:10.1371/journal.pone.0009645)

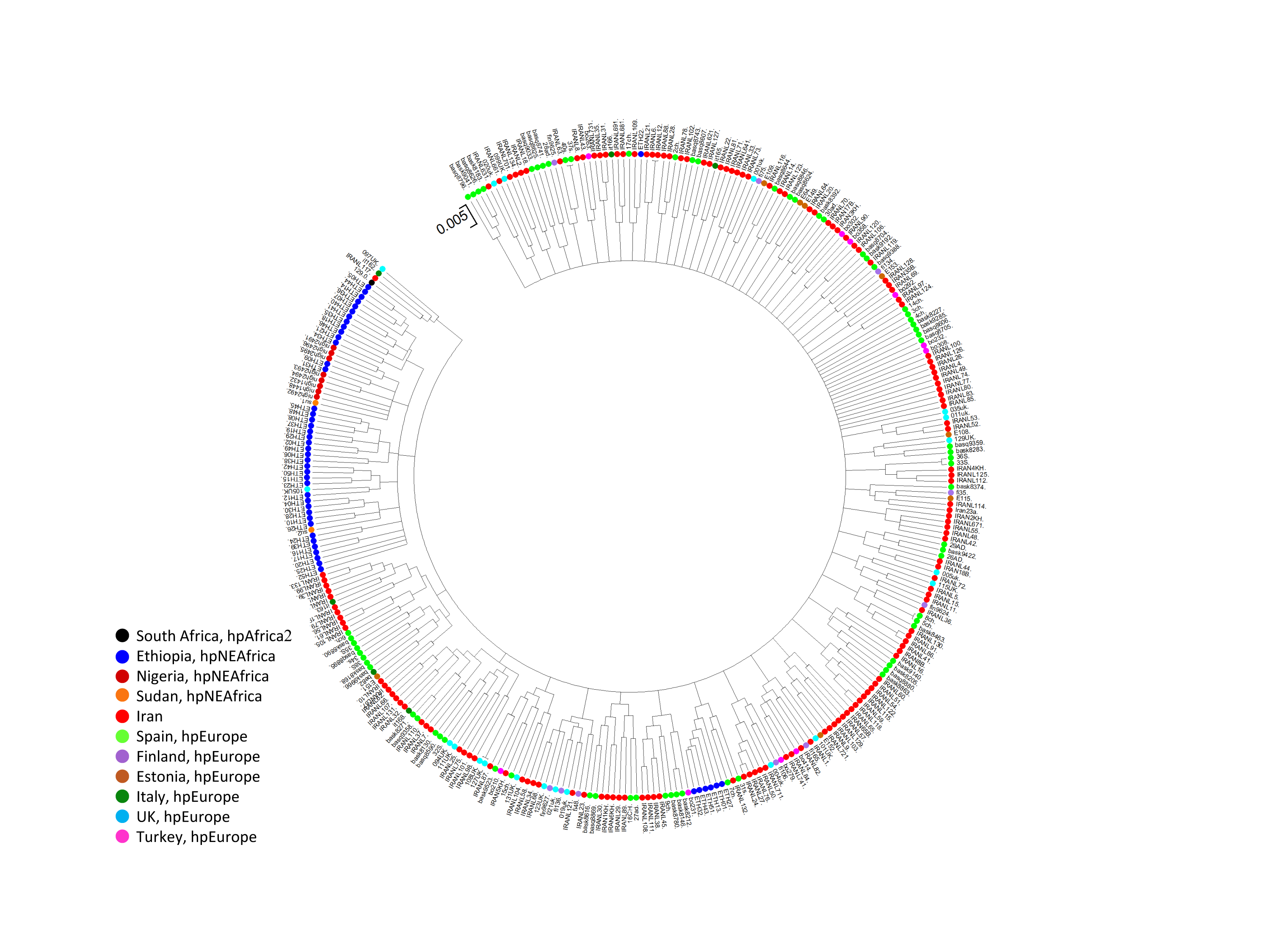

Supplement: Figure S1 — Phylogeny of 330 worldwide H. pylori strains using ClonalFrame. The majority-rule consensus tree showed a very close relationship between Iranian strains and European counterparts. Most European isolates, especially from Spain, UK, Finland and Italy shared most recent common ancestor with Iranian ones in different sub-clades. The strains from NEAfrica were also grouped into two distinct clades. The strains were colour-coded according to the origins they were isolated. (1.07 MB TIF) [file pone.0009645.s001.tif]
